# Salvage therapies of autoimmune hepatitis limit proinflammatory immune cells while sparing regulatory T cells

**DOI:** 10.1097/HC9.0000000000000088

**Published:** 2023-03-24

**Authors:** Finn C. Derben, Henriette Ytting, Björn Hartleben, Heike Bantel, Heiner Wedemeyer, Gro L. Willemoe, Elmar Jaeckel, Richard Taubert

**Affiliations:** 1 Department of Gastroenterology, Hepatology, Infectious Diseases and Endocrinology, Hannover Medical School, Hanover, Germany; 2 European Reference Network on Hepatological Diseases (ERN RARE-LIVER); 3 Department of Gastroenterology and Hepatology, Rigshospitalet, University of Copenhagen, Copenhagen, Denmark; 4 Department of Gastroenterology and Hepatology, Hvidovre University Hospital, Copenhagen, Denmark; 5 Institute for Pathology, Hannover Medical School, Hannover, Germany; 6 Department of Pathology, Rigshospitalet, University of Copenhagen, Copenhagen, Denmark; 7 Ajmera Transplant Center, Toronto General Hospital, United Health Network, University of Toronto, Toronto, Canada

## Abstract

Autoimmune hepatitis (AIH) can be clinically controlled by first-line immunosuppressive therapy in the majority of patients. However, a selective decrease in intrahepatic regulatory T cells (Treg) was observed with immunosuppressive therapy, which was even more pronounced in patients with incomplete responses than in patients who achieved biochemical remission. The effects of salvage therapies on the number of intrahepatic T and B cells, including Treg, are unclear. The hypothesis was that calcineurin inhibitors would further decrease intrahepatic Treg numbers, and the mammalian target of rapamycin inhibitors would increase intrahepatic Treg numbers.

**Methods:**

In this retrospective study at 2 centers, CD4^+^, CD8^+^ and CD4^+^FOXP3^+^ T cells, and CD79a^+^ B cells were quantified in surveillance biopsies under non–standard-of-care treatment [non-SOC: calcineurin inhibitor (n=10), second-line antimetabolites (n=9), mammalian target of rapamycin inhibitors (n=4)] compared with patients under the standard-of-care treatment (SOC).

**Results:**

Intrahepatic T-cell and B-cell counts were not significantly different between patients with biochemical remission under SOC and non-SOC. However, patients with incomplete response under non-SOC had significantly lower liver infiltration with T and B cells, whereas Treg were not reduced compared with SOC. This resulted in an even higher ratio of Treg to T and B cells in non-SOC compared with SOC when biochemical remission was not achieved. The different non-SOC regimens showed no significant difference in liver infiltration with T cells, including Treg and B cells.

**Conclusions:**

Non-SOC in AIH partially controls intrahepatic inflammation by limiting the hepatic infiltration of total T and B cells as the main drivers of inflammation without further decreasing intrahepatic Treg. A negative effect of calcineurin inhibitor and a positive effect of mammalian target of rapamycin inhibitors on the number of intrahepatic Treg was not observed.

## INTRODUCTION

Autoimmune hepatitis (AIH) is thought to result from a break of hepatic tolerance in genetically predisposed individuals triggered by external factors such as drugs or viral infections.^1^–^3^

Although regulatory T cells (Treg) enrich in actively inflamed livers of AIH patients without an overall numerical defect,^4^–^6^ several studies describe functional Treg impairments in AIH patients.^1^,^7^ Although some of these impairments resolve with therapy, the intrahepatic microenvironment of chronically inflamed livers appears to be deficient in the Treg-survival factor IL-2.^1^,^8^ IL-2 deficiency predisposes intrahepatic Treg to Fas-mediated apoptosis.^8^ This is consistent with the observation of increased apoptosis of Treg in AIH patients and may explain the observation of selective Treg decline under immunosuppressive therapy.^6^,^9^–^11^ Moreover, patients with incomplete remission (IR) under immunosuppressive therapy have lower intrahepatic Treg counts than patients who actually achieved biochemical remission (BR).^6^ Furthermore, higher baseline IL-2 serum levels in children with newly diagnosed AIH are associated with the achievement of BR on immunosuppressive therapy.^12^

To date, immunomodulatory therapies with exogenous IL-2 that have a beneficial effect on intrahepatic Treg^8^,^13^ and specific Treg cell therapies have been used only experimentally.^13^ However, salvage therapies with the best available evidence, mostly from retrospective or uncontrolled prospective studies, include second-line antimetabolites (2nd AM), for example, 6-mercaptopurine or mycophenolate, and calcineurin inhibitors (CNI), for example, ciclosporin or tacrolimus. 2nd AM are usually recommended for intolerance to the standard of care (SOC) and CNI for inadequate disease control with SOC.^14^ The least studied salvage agents are the mTOR inhibitors (mTOR-I), everolimus (EVR) and sirolimus.^15^–^17^ Although CNIs appear to attenuate Treg more than effector T cells (Teff), Treg are more robust to mTOR-I than to Teff.^18^ This is also supported by a study after liver transplantation, in which an increase in intrahepatic Treg was observed after switching from CNI to mTOR-I.^19^ Salvage therapies with CNI or mTOR-I are usually initiated in patients with IR after SOC, in whom the number of intrahepatic Treg is low anyway.^6^ Although both drug classes can clinically control the disease activity of AIH, the effects on intrahepatic immune regulation in terms of restoring hepatic tolerance are unknown.

The aim of the current study was to investigate the intrahepatic T-cell and B-cell compartment under ongoing salvage immunosuppressive therapies in comparison to SOC to address the question of whether local immune regulation through Treg is affected by non-SOC, hypothesizing that CNI reduces intrahepatic Treg count while mTOR-I increase it.

## METHODS

### Patients

We included patients with histologically proven AIH and with a surveillance liver biopsy under ongoing second-line or third-line therapy^14^ from 2 centers of the European Reference Network for Hepatologic Diseases (Hannover/Germany: n=13; Copenhagen/Denmark: n=10). Patient data during surveillance biopsy are summarized in Table 1. The data of the comparison cohorts under ongoing SOC with azathioprine and/or steroids were taken from a previous study.^6^

**TABLE 1:** Clinical and patient data at time of surveillance biopsy in AIH patients with non–standard of care

Clinical and patient data at the time point of surveillance biopsy	Calcineurin inhibitors	Second-line antimetabolite	mTOR inhibitors
No. of biopsies	10	9	4
Patient age at biopsy (y) median (range)	30 (17–53)	55 (32–73)	57 (43–69)
Sex (female/male), no. cases	7/3	6/3	3/1
AIH type 1/2, no. cases	5/1, n=6	8/1	4/0
Non–standard-of-care therapy at time of biopsy, no. cases (%)			
Ciclosporin/tacrolimus	1 (10%)/6 (60%)	—	—
Ciclosporin/tacrolimus + mycophenolate mofetil	1 (10%)/2 (20%)	—	—
Mycophenolate mofetil	—	6 (67)	—
Mercaptopurine	—	3 (33)	—
Everolimus/everolimus + MMF	—	—	2 (50%)/2 (50%)
Coimmunosuppression at time of biopsy, no. cases, n (%)	9 (90)	7 (78)	4 (100)
Glucocorticoid (Predniso(lo)n/budenoside), n (%)	1 (10)	7 (78)	—
Azathioprine, n (%)	2 (20)	—	—
Glucocorticoid + azathioprine, n (%)	3 (30)	—	2 (50)
Glucocorticoid + 2nd-line antimetabolite, n (%)	3 (30)	—	2 (50)
Dosage (mg), range			
Ciclosporin	50–200	—	—
Tacrolimus	2–6	—	—
Mycophenolate mofetil	1500–2000	250–2000	500–1000
Mercaptopurine	—	37.5–150	—
Everolimus	—	—	1.5–3.0
Immunsuppression level at time of biopsy (ng/mL), range			
Tacrolimus	4.4–6.0	—	—
Ciclosporin	92–135	—	—
Everolimus	—	—	2.5–5.8
Duration of SOC/non-SOC at time of biopsy (months), median (range)	42 (3–110)	50 (3–86)	45 (34–55)
Reason switch to non-SOC, no. cases, n (%)			
Incomplete response	9 (90)	3 (33)	3 (75)
Intolerance/side effects	1 (10)	6 (66)	1 (25)
Laboratory tests at time of biopsy (times upper limit of normal), median (range)			
Alanine aminotransferase	3.5 (0.8–16.5)	1.2 (0.4–32.9)	1.0 (0.7–48.0)
Alkaline phosphatase	0.9 (0.6–2.5), n =5	0.8 (0.5–3.2), n =7	1.0 (0.6–94.0)
Bilirubin	1.0 (0.6–2.9), n =5	0.7 (0.3–5.4), n =8	0.4 (0.3–8.0)
IgG	1.2 (0.6–3.0), n =9	0.9 (0.7–1.0), n =7	1.1 (0.7–8.6), n =3
Histology, median (range)			
mHAI	7.5 (3–13)	3 (1–10)	1 (1–4), n=3
Stage of fibrosis (Ishak F)	4 (0–6)	1 (0–6)	0 (0–1), n=3

Abbreviations: AIH, autoimmune hepatitis; mHAI, modified hepatitis activity index; mTOR, mammalian target of rapamycin inhibitors; non-SOC, non–standard of care; SOC, standard of care.

This study was approved by the local research ethics committee of Hannover Medical School. Written informed consent was obtained from all patients from the prospective biomaterial repository of Hannover Medical School (approval number 5582). The use of material and data from external patients in this cohort from 2 centers was approved by the respective local ethics committees. This study was performed in accordance with both the Declarations of Helsinki and Istanbul.

### Histology

Biopsies were processed, and histologic evaluation for the modified hepatitis activity index (mHAI) and fibrosis was performed by an experienced liver pathologist in a blinded manner, as published.^6^,^9^

**FIGURE 1:**
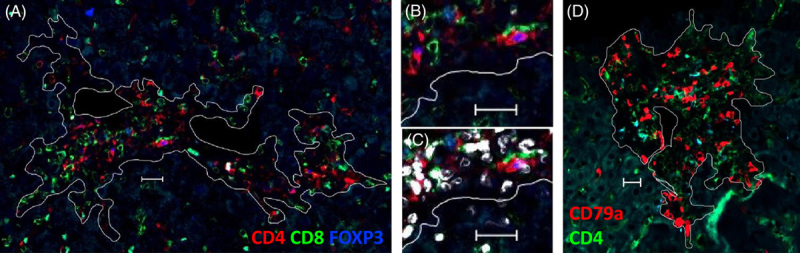
Multicolor immunofluorescence of human liver biopsies. (A) T-cell staining in a single formalin-fixed and paraffin-embedded liver biopsy section and (D) B-cell staining in subsequent liver biopsy section of an autoimmune hepatitis patient under non–standard-of-care therapy. White lines surround the evaluated area of portal infiltrates and exclude the lumen of veins, arteries, and bile ducts. (B) Surface expression of CD4 (red) and CD8 (green) in comparison to (C) nuclear colocalization of FOXP3 (blue) and DAPI (white). White bars represent 20 µm.

Immunofluorescence staining of liver-infiltrating lymphocytes in formalin-fixed and paraffin-embedded liver biopsies was performed as described.^6^,^9^ The intrahepatic infiltration density of CD4^+^CD8^−^ T cells, CD8^+^CD4^-^ T cells, CD4^+^CD8^−^FOXP3^+^ Treg, and CD79a^+^ (Pan–B-cell marker) in portal-derived liver infiltrates (Figure 1) was quantified as recently described.^6^,^9^ CD4^+^ T cells were distinguished from CD4^weak^ liver sinusoidal endothelial cells on the basis of their cell morphology, localization, and intensity of CD4 expression. Histological quantification of the frequency of CD4^+^CD8^−^FOXP3^+^ Treg compared with the number of CD4^+^ and CD8^+^ T cells was validated by the demethylation status of the FOXP3 locus, which is specifically demethylated in Treg but not in activated Teff, and by flow cytometry in previous studies.^6^,^20^ In addition, CD8^+^CD4^−^FOXP3^+^ T cells, representing the activated cytotoxic T cells, were quantified as a measure of contamination of activated Teff within the pool of FOXP3^+^ cells. In this study, only 7.0% of FOXP3^+^ cells were CD8^+^, underscoring that the histologic method detects Treg rather than activated Teff.

### Statistical analysis

Statistical analysis was performed using SPSS 15.0 and GraphPad Prism 5. Comparisons of more than 2 groups were performed using ANOVA with the Dunnett *post hoc* test for all continuous variables. Semiquantitative histological scores were compared using the Kruskal-Wallis test with Dunn *post hoc* analysis. *p*-values<0.05 (double-tailed) were considered statistically significant in all the analyses.

## RESULTS

We were able to retrospectively recruit 23 AIH patients who underwent surveillance biopsies while on ongoing salvage therapy at 2 centers. Patient data are shown in Tables 1 and 2 and Supplemental Table 1 (http://links.lww.com/HC9/A207). Of these 23 AIH patients, 15 were switched to nonstandard therapy (non-SOC) because of IR under SOC with azathioprine and/or steroids, whereas 9 patients were intolerant to SOC. The majority of patients with IR on SOC (12/15) were switched to CNI or EVR, and the majority of patients with SOC intolerance (6/9) were switched to a 2nd AM.

**TABLE 2:** Clinical and patient data at the time of surveillance biopsy in AIH patients with standard and non–standard of care according to the treatment response

	Biochemical remission		Incomplete response	
Clinical and patient data at the time point of surveillance biopsy	SOC	Non-SOC	SOC	Non-SOC
No. biopsies	16	6	11	17
Patient age at biopsy (y,) median (range)	49 (20–66)	58 (43–73)	48 (26–66)	42 (17–65)
Sex (female/male), number of cases	8/8	6/0	10/1	10/7
AIH type 1/2, number of cases	15/1	5/1	11/0	12/1
Non–standard-of-care therapy at time of biopsy, no. cases, n (%)				
Ciclosporin/tacrolimus	—	1 (17%)/0 (0%)	—	0 (0%)/6 (35%)
Ciclosporin/tacrolimus + mycophenolate mofetil	—	0 (0%)/0 (0%)	—	1 (6%)/2 (12%)
Mycophenolate mofetil/mercaptopurine	—	1 (17%)/2 (33%)	—	5 (30%)/1 (6%)
Everolimus/everolimus + MMF	—	1 (17%)/1 (17%)	—	1 (6%)/1 (6%)
Coimmunosuppression at time of biopsy, no. cases, n (%)				
Glucocorticoid (predniso(lo)n/budenoside)	9 (56)	—	10 (91)	—
Azathioprine	13 (81)	—	8 (73)	—
Glucocorticoid + azathioprine	6 (38)	—	7 (64)	—
Glucocorticoid + 2nd-line antimetabolite	—	—	—	—
Dosage (mg), range				
Ciclosporin	—	200	—	50
Tacrolimus	—	—	—	2–6
Mycophenolate mofetil	—	250–500	—	1000–2000
Mercaptopurine	—	37.5–50	—	150
Everolimus	—	0.5–3.0	—	1.5–3.0
Immunosuppression level at time of biopsy (ng/mL), range				
Tacrolimus	—	—	—	4.4–6.0
Ciclosporin	—	135	—	92
Everolimus	—	2.6–3.9	—	2.5–5.8
Duration of SOC/non-SOC at time of biopsy (mo), median (range)	41 (6–144)	58 (34–110)	45 (9–157)	36 (3–84)
Reason for switch to non–standard-of-care therapy, no. cases, n (%)				
Incomplete response	—	3 (50)	—	12 (71)
Intolerance/side effects	—	3 (50)	—	5 (29)
Laboratory tests at time of biopsy (times ULN), median (range)				
Alanine aminotransferase	0.6 (0.3–1.0)	0.7 (0.4–0.9)	2.0 (0.4–14.3)	2.6 (0.7–48.0)
Alkaline phosphatase	0.5 (0.3–0.7); n=14	0.6 (0.6–0.8); n=5	1.0 (0.4–2.5)	1.2 (0.5–9.4), n=11
Bilirubin	0.6 (0.3–2.1), n=15	0.4 (0.3–0.6), n=5	0.8 (0.3–3.7), n=10	1.0 (0.4–8.0), n=12
IgG	0.8 (0.4–1.0), n=6	0.7 (0.7–0.8), n=3	0.9 (0.6–1.9), n=7	1.0 (0.6–3.0), n=15
Histology, median (range)				
mHAI	1.5 (0–5)	2 (1–6)	6 (2–10)	5.5 (1–13)
Stage of fibrosis (Ishak F)	0 (0–5)	0.5 (0–2)	1 (0–6)	3 (0–6)

Abbreviations: AIH, autoimmune hepatitis; mHAI, modified hepatitis activity index; non-SOC, non–standard of care; SOC, standard of care; ULN, upper limit of normal.

The history of immunosuppression in these patients was as heterogeneous as the ongoing therapy at the time of the liver biopsies analyzed in this study (Tables 1 and 2 and Supplemental Table 1, http://links.lww.com/HC9/A207). To allow a structured analysis, patients treated with CNI and patients receiving combination therapy with CNI and 2nd AM were pooled in the CNI group (n=10). Similarly, patients treated with EVR and 2nd AM were pooled in the EVR group (n=4). Only those who received only 2nd AM with and without steroids were included in the 2nd AM group (n=9) (Table 1).

Infiltration of the AIH liver by effector and Treg differed significantly between patients in BR and those in IR on immunosuppressive therapy.^6^ Therefore, the response to treatment under ongoing non-SOC should also be included in the analysis of liver-infiltrating lymphocytes. Because of the small sample size, the high variability, and the overlap in the non-SOC groups, a combined analysis, including both factors, type of non-SOC, and response to treatment (BR or IR), was not reasonable. Therefore, we chose a stepwise approach.

**FIGURE 2:**
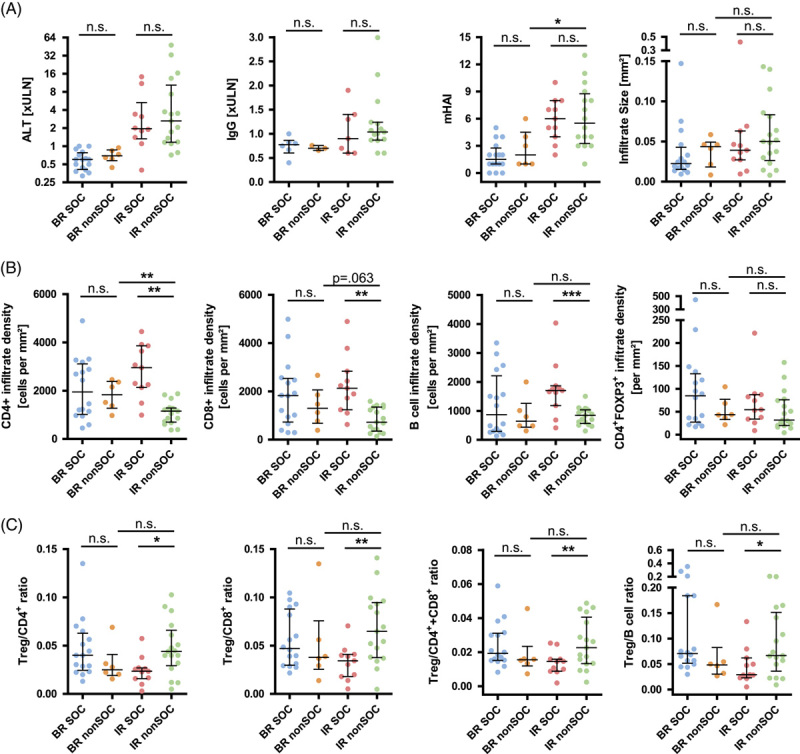
Control of autoimmune hepatitis (AIH) disease activity under standard and non–standard of care. (A) Comparison of serological markers of disease activity [alanine aminotransferase (ALT) at the time of biopsy and modified hepatitis activity index (mHAI) and size of liver infiltrates as histological parameters between AIH patients with standard of care therapies (SOC) and AIH patients with non–standard of care (non-SOC) according to the treatment response (biochemical remission, BR, incomplete response, IR]. (B) The density of liver infiltration with CD4^+^ and CD8^+^ T cells, CD79a^+^ B cells, and CD4^+^FOXP3^+^ regulatory T cells (Treg) and cell ratios of Treg to T and B cells (C). (Horizontal lines represent median and error bars represent the interquartile range; *p*≥0.05; *p<0.05; **p<0.01; ***p<0.001.) Abbreviations: n.s., not significant; ULN, upper limit of normal.

First, the basal clinical data (Figure 2A) and liver-infiltrating lymphocytes (Figure 2B, C) of patients with non-SOC were compared with those of SOC, according to the achievement of BR and IR (Table 2). In addition, patients with BR under non-SOC were compared with those with IR under SOC. The comparison of patients with BR and IR under SOC was performed in a previous study.^6^ According to the criteria of the current international guidelines, only 6/23 patients (26%) under non-SOC were in BR at the time of the surveillance biopsies. The data of AIH patients with SOC were taken from our previous study.^6^ As expected, ALT and IgG levels did not differ significantly between SOC and non-SOC because both parameters were used to define the treatment response (BR and IR). The histologic disease severity, as measured by mHAI, and the size of portal-based infiltrates in the liver also did not differ between SOC and non-SOC within each treatment response group. Patients with IR under non-SOC had a higher mHAI than those who achieved BR under non-SOC (Figure 2A).

When BR was achieved under non-SOC, infiltration of the liver by CD4^+^ and CD8^+^ T cells, CD79a^+^ B cells, and CD4^+^FOXP3^+^ Treg was not significantly different from AIH patients who achieved BR under SOC (Figure 2B). In contrast, patients with IR under non-SOC had lower infiltration density of CD4^+^ and CD8^+^ T cells and CD79a^+^ B cells than AIH patients with IR under SOC. Although BR was not achieved, liver infiltration with CD4^+^ T cells was even lower in AIH patients with IR under non-SOC than in those with BR under non-SOC. Interestingly, liver infiltration with CD4^+^FOXP3^+^ Treg cells did not differ significantly between SOC and non-SOC within each treatment response group (BR and IR) nor between BR under non-SOC and IR under non-SOC (Figure 2B). Lower infiltration with T and B cells overall and unaffected infiltration with Treg cells resulted in higher Treg frequencies in patients with IR under non-SOC compared with IR under SOC, whereas there were no significant differences in Treg frequencies when BR under non-SOC and IR under non-SOC were compared (Figure 2C).

**FIGURE 3:**
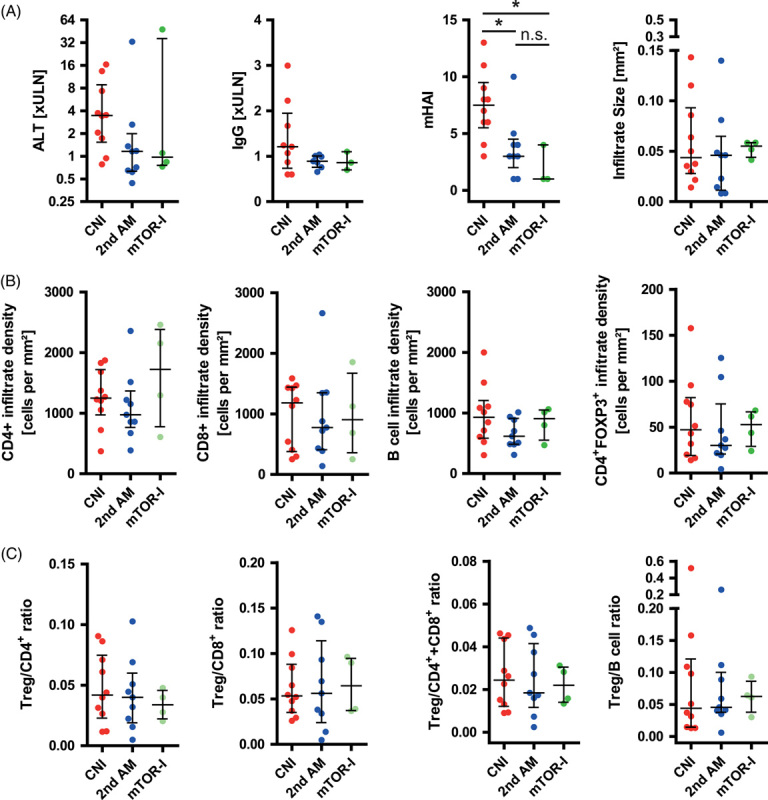
Control of autoimmune hepatitis (AIH) disease activity under different regimen of non–standard of care. (A) Comparison of serological markers of disease activity [alanine aminotransferase (ALT) at time point of biopsy, as well as modified hepatitis activity index (mHAI) and size of liver infiltrates as histological parameters between AIH patients with different regimen of non–standard of care (second-line antimetabolites, 2nd AM; calcineurin inhibitors, CNI; mTOR inhibitors, mTOR-I]. (B) The density of liver infiltration with CD4^+^ and CD8^+^ T cells, CD79a^+^ B cells, and CD4^+^FOXP3^+^ regulatory T cells (Treg) and cell ratios of Treg to T and B cells (C). (Horizontal lines represent median and error bars represent the interquartile range *p*≥0.05; *p<0.05; nonsignificant differences were not outlined.) Abbreviations: mTOR, mammalian target of rapamycin inhibitors; n.s., not significant; ULN, upper limit of normal.

Subsequently, the same clinical data and liver-infiltrating lymphocyte parameters were compared between the different treatment regimens (CNI, 2nd AM, and mTOR-I) within the non-SOC cohort, regardless of the treatment response (Figure 3; Table 1). The serum parameters of disease activity (ALT, IgG) did not differ between the 3 non-SOC therapies (Figure 3A). Although the size of liver infiltrates did not differ among the 3 non-SOC therapies, mHAI was higher in CNI-treated patients and did not differ between 2nd AM and mTOR-I. None of the liver-infiltrating lymphocyte parameters examined (infiltration density and cell percentage) differed significantly between the 3 non-SOC treatment groups (Figure 3B, C).

## DISCUSSION

Immunosuppressive therapy for AIH has dramatically improved life expectancy and slowed disease progression, especially in patients who achieve BR.^21^ However, the current immunosuppressive therapies seem to control but not reverse autoimmunity, resulting in a high relapse rate after the discontinuation of medication and, in most patients, lifelong dependence on immunosuppressive therapy. Mechanistically, persistent resistance of autoimmune T cells to therapy and a numerical decrease in intrahepatic Treg may provide at least some immunologic explanations for these clinical observations.^6^,^9^,^10^,^22^ The association of fewer intrahepatic Treg in patients with IR raised the question of whether clinically applied salvage therapies might help restore the local immune regulation or, in the case of CNI, further attenuate the intrahepatic Treg.

This study is the first analysis of intrahepatic lymphocytes under ongoing salvage therapies in AIH. The patient histories of this cohort are unique and highly heterogeneous. Half of the patients who achieved BR under non-SOC had a 2nd AM, and 50% were switched from SOC to non-SOC because of intolerance (Tables 1 and 2). In contrast, patients who did not achieve BR under non-SOC were switched to non-SOC in 70% of cases because they did not respond completely to SOC. Although <20% of patients with BR under non-SOC were treated with CNI, over 50% of patients with IR under non-SOC received CNI. Thus, as expected, the cohort of continued IR under non-SOC was more enriched with difficult-to-treat AIH cases than the cohort of BR under non-SOC. The doses of 2nd AM were lower in patients who achieved BR than in those with IR. Trough levels of CNI and mTOR-I were within an intermediate dosing range in all non-SOC groups.

The treatment duration of 3–5 years from the start of SOC or non-SOC to surveillance biopsy was long enough to expect the distinct histopathologic effects of the respective treatment regimen. In contrast to the patients with SOC, the patients with non-SOC had already undergone a previous treatment period with SOC of months to years (Supplemental Table 1, http://links.lww.com/HC9/A207). Thus, patients with non-SOC were seen per se after a longer overall treatment duration and after a longer disease course. In addition, patients with CNI were younger (median 30 y) than the other non-SOC patients (median 55–57 y).

Contrary to our initial hypothesis, we did not observe a further decrease in intrahepatic Treg numbers under CNI, nor an increase in Treg numbers under mTOR-I. Moreover, the lower Treg frequency in the liver during IR compared with BR observed in our previous study was not found under non-SOC.^6^ This could be related, at least in part, to the large heterogeneity of the cohort. This lack of difference between IR and BR patients under non-SOC is interesting because the total number of CD4 and CD8 T cells, which are the main source of IL-2 in the inflamed liver,^8^ was even lower in IR under non-SOC. However, we can only speculate about the mechanistic background because further immunologic analyses were not possible in this retrospective study with rare and limited patient material. Similarly, in this retrospective analysis, blood samples were not available to quantify cytokines to investigate the hypothesis of IL-2 deficiency or to quantify Treg in peripheral blood.

The lower infiltration with T and B cells in IR under the more intensive non-SOC compared with BR under non-SOC argues for partial control of liver inflammation, even in patients with IR. This putative better control of T and B-cell infiltration with stable Treg infiltration in IR patients under non-SOC surprisingly resulted in higher cell ratios of Treg to total T and B cells compared with IR patients under SOC.

In summary, the original hypothesis of a lower intrahepatic Treg count in CNI-treated and a higher intrahepatic Treg count in mTOR-treated AIH patients was not confirmed in this cross-sectional study with rather heterogeneous patients. An analysis of paired biopsies before and after conversion from CNI-based to mTOR-I–based immunosuppression revealed an increased number of intrahepatic Treg in liver transplant recipients.^19^

Murine models of AIH could fill the mechanistic gaps of this limited study with human samples and help understand the basic immunologic effects of non-SOC on AIH. Although murine models have been used to study the effects of less commonly used salvage therapies, such as rituximab and low-dose IL-2 administration, data on the effects of CNI or mTOR-I in murine AIH models are unfortunately lacking to date. In contrast, we were unable to recruit sufficient numbers of AIH patients with available surveillance biopsies under ongoing B-cell depletion or low-dose IL-2 therapy to investigate this in patients.

The results of this study must be interpreted with caution because the patient numbers were small, the cohorts were heterogeneous, and the patients’ medical histories were long, unique, and hardly comparable. In addition, non-SOC therapies partially overlapped. Patients requiring salvage therapy generally represent a sample of patients with the most aggressive disease course. We tried to avoid overanalysis of the data by reducing statistical comparisons to those necessary to answer the hypothesis of this study. In addition, paired longitudinal liver biopsies at IR under SOC and later under non-SOC would have been more informative. However, the number of patients who could be recruited for this study must be viewed in the context that AIH is a rare disease that generally responds well to treatment. Only a minority of AIH patients require a change in treatment to non-SOC therapy, and only a minority of patients receiving non-SOC therapy undergo a rebiopsy to assess the disease control. Less than 20 AIH cases of mTOR-I therapy with and without surveillance biopsies have been published, and one fifth of these valuable patients could be included in this study.^15^–^17^ Nevertheless, further studies with larger cohorts, at best with paired biopsies before and after a switch from SOC to non-SOC, need to confirm the results of this retrospective pilot study.

Technical limitations of the study are the limited number of markers used to define Treg and Teff, which also express FOXP3 after activation. We validated histological quantification using epigenetic analysis of the Treg-specific demethylation region of the FOXP3 gene and flow cytometry, including additional markers such as CD25 and CD127,^6^,^20^,^23^–^25^ which could not be quantified in paraffin-embedded tissue at the start of this project. Although only a minority of FOXP3^+^ cells in this study were activated CD8^+^FOXP3^+^ Teff (7%), we cannot exclude relevant contamination of CD4^+^FOXP3^+^ cells, referred to here as Treg, by activated T helper cells. The limited number of markers used here also prevented the assessment of subsets of CD8 T cells or B cells infiltrating the liver. More advanced technologies, such as single-cell sequencing of the liver-infiltrating lymphocytes, could overcome the limitation of limited marker sets but could not be applied in retrospectively recruited, paraffin-embedded tissue. Another limitation is the lack of comparison with normal and healthy liver tissue as a comparison cohort.

In conclusion, the disease activity of AIH and its key intrahepatic factors, T and B cells, are partially controlled under non-SOC, with no evidence of further imbalance in intrahepatic Treg-mediated immune regulation. The recently observed lower intrahepatic Treg counts in patients with IR compared with patients with BR could not be confirmed under non-SOC. The international multicenter registries with information on local biorepositories, such as the European Reference Network Rare-Liver and the International AIH Group, will facilitate further analysis of such rare but valuable AIH patient cohorts in the future.

## Supplementary Material
