# Supplementary material for: Salvage therapies of autoimmune hepatitis limit proinflammatory immune cells while sparing regulatory T cells
Source: Hepatol Commun. 2023 Mar 24;7(4):e0088. doi: 10.1097/HC9.0000000000000088 (PMC10043582; doi:10.1097/HC9.0000000000000088)
Supplement: Supplementary file 1 [file hc9-7-e0088-s001.docx]

**Supplemental Material**

**Supplemental Table 1**

| **Calcineurin inhibitors** | **Initial immunosuppression** | **Duration initial therapy (months)** | **nonSOC therapy agent** | **Co-immunosuppression** | **Duration nonSOC therapy until biopsy (months)** |
| --- | --- | --- | --- | --- | --- |
| CNI1 | n/a | 19 | Calcineurin inhibitor | Glucocorticoid | 110 |
| CNI2 | Glucocorticoid + SOC-antimetabolite | 12 | Calcineurin inhibitor | - | 48 |
| CNI3 | Glucocorticoid | - | Calcineurin inhibitor | Glucocorticoid + SOC-antimetabolite | 8 |
| CNI4 | Glucocorticoid + SOC-antimetabolite | 24 | Calcineurin inhibitor + 2nd line antimetabolite | Glucocorticoid | 72 |
| CNI5 | Glucocorticoid + SOC-antimetabolite | 12 | Calcineurin inhibitor | Glucocorticoid + SOC-antimetabolite | 4 |
| CNI6 | Glucocorticoid + SOC-antimetabolite | 168 | Calcineurin inhibitor + 2nd line antimetabolite | Glucocorticoid | 80 |
| CNI7 | Glucocorticoid + SOC-antimetabolite | 120 | Calcineurin inhibitor | Glucocorticoid + SOC-antimetabolite | 3 |
| CNI8 | Glucocorticoid + SOC-antimetabolite | 72 | Calcineurin inhibitor | SOC-antimetabolite | 84 |
| CNI9 | Glucocorticoid + SOC-antimetabolite | 22 | Calcineurin inhibitor | SOC-antimetabolite | 36 |
| CNI10 | Glucocorticoid + SOC-antimetabolite | 189 | Calcineurin inhibitor + 2nd line antimetabolite | Glucocorticoid | 19 |
| **2nd line antimetabolite** |  |  |  |  |  |
| AM1 | n/a | 29 | 2nd line antimetabolite | Glucocorticoid | 63 |
| AM2 | Glucocorticoid | 6 | 2nd line antimetabolite | Glucocorticoid | 35 |
| AM3 | Glucocorticoid + SOC-antimetabolite | 7 | 2nd line antimetabolite | - | 61 |
| AM4 | Glucocorticoid + SOC-antimetabolite | - | 2nd line antimetabolite | Glucocorticoid | 50 |
| AM5 | n/a | 21 | 2nd line antimetabolite | - | 86 |
| AM6 | n/a | 33 | 2nd line antimetabolite | Glucocorticoid | 3 |
| AM7 | Glucocorticoid | 9 | 2nd line antimetabolite | Glucocorticoid | 36 |
| AM8 | Glucocorticoid | 189 | 2nd line antimetabolite | Glucocorticoid | 3 |
| AM9 | n/a | 97 | 2nd line antimetabolite | Glucocorticoid | 54 |
| **Everolimus** |  |  |  |  |  |
| EV1 | Glucocorticoid + SOC-antimetabolite | 120 | Everolimus + 2nd line antimetabolite | Glucocorticoid | 55 |
| EV2 | Glucocorticoid + SOC-antimetabolite | 396 | Everolimus | Glucocorticoid + SOC-antimetabolite | 50 |
| EV3 | Glucocorticoid + SOC-antimetabolite | 204 | Everolimus | Glucocorticoid + SOC-antimetabolite | 39 |
| EV4 | Glucocorticoid + SOC-antimetabolite | 72 | Everolimus + 2nd line antimetabolite | Glucocorticoid | 34 |
